# A Symposium on the Pathology and the Treatment of Typhoid Fever, with Special Reference to the So-Called Antiseptic, Eliminative, Abortive, or Woodbridge Treatment

**Published:** 1902-05

**Authors:** James B. Baird

**Affiliations:** Atlanta, Ga.


ATLANTA

### Journal-Record of Medicine.

**Successor to Atlanta Medical and Surgical Journal, Established 1855, and Southern Medical Record, Established 1870.**

VOL. IV. MAY, 1902. No. 2.

BERNARD WOLFF, M.D., EDITOR, Nos. 319–20 Prudential.

Published Monthly.

M. B. HUTCHINS, M.D., BUSINESS MANAGER, No. 64 Marietta St.

### ORIGINAL COMMUNICATIONS.

### A SYMPOSIUM ON THE PATHOLOGY AND THE TREATMENT OF TYPHOID FEVER, WITH SPECIAL REFERENCE TO THE SO-CALLED ANTISEPTIC, ELIMINATIVE, ABORTIVE, OR WOODBRIDGE TREATMENT.*

BY JAMES B. BAIRD, M.D.,

ATLANTA, GA.

The commonly accepted doctrine of the day ascribes typhoid fever to a specific cause—to the presence, and to the resultant effects, within the body of the typhoid bacillus.

If this theory of causation is undoubtedly correct, or, rather, if the disease is, in fact, as some would have us believe, dependent solely upon the mere presence of the special germ, and if the only habitation of the germ in the organism is the alimentary tract, the reasons advanced in favor of the eliminative and antiseptic—

* Read before the Medical Association of Georgia, at Savannah, April 16, 1902.

the so-called Woodbridge, abortive treatment, with its numerous amendments, modifications and variations—may afford a rational basis for the support of a system of treatment designed to abate this simple and uncomplicated deviation from normal conditions.

If, however, upon the other hand, the exact cause and the precise pathological changes are not clearly established, and are not shown to lie within the easy reach of cathartics and of intestinal antiseptics, we shall be compelled to defer the acceptance of any aggressive mode of management, based upon purely theoretical grounds, or upon mere assumption only, and must patiently await the day when pathologists and careful clinical observers shall have removed some of the confusing obscurity which now appears to invest a complicated subject.

We shall see, as we proceed, what the pathologists of the present time really believe.

Meanwhile, as the disease actually exists, and more or less vigorously asserts itself year after year, the duty and the obligation to determine the best mode of meeting its frequent invasions rest upon us, and our responsibility to our patients justifies—yea, demands—a careful review and a patient consideration of this important question.

If the established principles of treatment are best, they should not be departed from. If, on the contrary, more recent methods—whether they can be sustained by logical reasoning or not—should be shown by experience of ample scope and of trustworthy kind to offer any improvement, or to promise any advantage, they certainly should not be disregarded or lightly cast aside.

How, then, can practical conclusions be reached? It must be admitted, for obvious reasons, that the comparatively limited experience of an individual cannot be accepted as conclusive, and that no amount of argument can, in the nature of things, establish the truth, but out of the mouths of a multitude of competent witnesses substantial facts, worthy of credence, may be evolved.

Therefore, for this purpose, I have sought to procure such evidence, and I now have the privilege of presenting to you the concise views as to the pathology and the best manner of managing this disease, according to our present knowledge, of a number of the most accomplished and prominent pathologists and physicians in this country, consulted by me without any previous knowledge of their opinions, and without the slightest intention or the remotest desire on my part of influencing or seeking, in any wise, to bias the testimony which is herewith confidently submitted for your consideration, unaccompanied by further elaboration or comment from me.

Dr. T. M. Prudden, New York, thinks that the evidence is entirely conclusive that the Eberth bacillus is the inciting factor in typhoid fever.

Dr. L. Hectoen, Chicago, expresses the opinion that the agglutination reaction, and the demonstration of bacillus typhosus in the blood of typhoid fever cases are, to his mind, positive evidence of the etiological relationship of the bacillus to the disease.

Dr. A. P. Ohlmacher, Chicago, believes that the typhoid bacillus is the active, exciting cause in typhoid fever. The uniformity with which this organism is encountered in the organs chiefly affected is very strong evidence. To know the bacillus is pathogenic from experimental demonstration and its failure to incite typhoid in animals must be looked upon as due to natural immunity. While it does not induce typical experimental typhoid in animals, it is capable of provoking peritonitis, meningitis, etc., thus attesting its pathogenicity. Further evidence is afforded by the response of the bacillus to the agglutinins of the blood in recently immune human beings and of animals made immune. The burden of our testimony to the present time is to indicate the absolute specificity of the agglutinins. So, while he is personally satisfied as to the causal relationship of the typhoid bacillus, he is by no means clear as to the biological position of the organism. It belongs to the great typhoid-colon group of bacteria, a closely related group of intestinal bacilli, whose differential and specific characteristics are not yet clearly understood.

Dr. Simon Flexner, Philadelphia, says, the evidence is much better now than ever before that the typhoid bacillus is the cause of typhoid fever. This improvement has come from the discovery of the Gruber-Widal reaction. All the indications point to its being specific; thus confirming the relation of the typhoid bacillus to typhoid fever.

There is, however, another point that should be taken into consideration, namely, that there may be a disease resembling typhoid fever, and yet different from it, due to another micro-organism. Several such cases have already been reported in which the para-colon bacillus—in his opinion an unfortunate name—seems to be the causative agent. Whether we are justified in separating these two groups of cases the future only can decide. Thus far, para-colon infections have been discovered clinically only, no pathological findings have been secured. Durham's view is interesting and worth considering. It is that some of the cases of typhoid fever without intestinal lesions may be atypical and, perhaps, para-colon infections. He knows this is not true in every instance, for in one case of typhoid fever he found undoubtedly the bacillus typhosus without intestinal lesions. His confidence in the etiological role of the typhoid bacillus is complete, but he recognizes the possibility of a group of symptoms resembling typhoid fever being caused by an allied but different micro-organism.

Dr. H. F. Harris, Atlanta, says, since the enunciation of Koch's well known postulate, it has been universally conceded by pathologists and bacteriologists that in order to positively determine that a given micro-organism stands in a causal relationship to a disease it is necessary to comply with all of the conditions of this law. This has not been done in the case of Eberth's bacillus, since the lower animals do not have typhoid fever, and this investigation has not as yet been tested upon human beings. The injection of this bacillus into animals produces more or less pathological alterations within their bodies, but in no instance has it developed anything resembling typhoid fever. The colon bacillus, of which Eberth's bacillus appears to be only a variety, is likewise capable of producing similar changes when injected into living animal tissues. There are two principal reasons why scientific men have been disposed to accept the etiological relationship of this micro-organism to the disease. These are the usual presence of this germ in the lesions of typhoid fever, and the so-called Widal reaction. As regards the former, he asserted from personal observation that in some instances of typhoid fever the most careful examination of ulcers fails to reveal the presence of this particular bacillus, though it must be admitted that a few of them might be present and be overlooked. As before said, this germ appears to be but a variety of the colon bacillus, which organism is almost alalways present in the intestinal tract of human beings. As is well known, this bacillus shows a remarkable disposition to vary its form and cultural peculiarities under different conditions, and he would suggest that Eberth's bacillus might, after all, be nothing more than a slightly altered colon bacillus resulting from the new conditions that this organism is subjected to when growing and developing on an ulcerated surface. This might also account for the fact that many observers have isolated organisms indistinguishable from the typhoid bacillus in lesion of other parts of the body, and may explain the extraordinary resemblance between this so-called typhoid bacillus and the germ discovered by Shiga in dysenteric discharges.

The Widal test, as is known to every one, consists in subjecting cultures of certain bacteria, either dead or alive, to the action of the sera obtained from an individual suffering from a pathological state induced by similar germs, and under such conditions, the organisms experimented with, after a variable time, collect together in small clumps.

It is true that errors may occur in carrying out this test. He has seen many instances in which typical reactions were obtained from the blood of persons entirely well, and who had never suffered from typhoid fever, and on the other hand, he has seen cases where the sera of individuals unquestionably suffering from this disease failed entirely to produce any effect on Eberth's bacilli. In this connection it may not be inappropriate to call attention to the fact that many observers have obtained this reaction in a perfectly characteristic manner in cases of yellow fever with Sanarelli's bacillus icteroides,—which the investigation of Reed and Carrol conclusively show have nothing to do with this disease. However, there can be no question that this reaction is obtained in a greater or less degree in a majority of the cases of typhoid fever, and this should occasion no surprise when we consider that Eberth's bacillus is not only in the intestinal lesions, but quickly finds its way to the spleen and abdominal lymphatics of the affected individual, and the organisms growing and developing in the body very naturally produce changes in the blood that make it capable of causing the peculiar clumping which is the characteristic feature of this test. If this suggestion is a correct explanation of the presence of Eberth's bacillus in the lesions of typhoid fever, it would account for the failure to obtain the Widal reaction in some cases of this disease, because individuals are occasionally encountered in whose intestinal tracts no colon bacilli are present, and under such circumstances Eberth's bacillus would, of course, be absent, and as a consequence the blood of the individual would not give the serum test. This would also make clear the reason for mildness of typhoid fever in some instances and its great severity in others.

While he has no fixed views upon the subject, he suggests the possibility that typhoid fever may not be due to Eberth's bacillus, but is produced by some other pathogenic agent, and after the lesions are established, colon bacilli secondarily infect the diseased parts and become distributed throughout the entire body to a greater or less extent, producing decided pathological alteration, and adding greatly to the severity of the disease. The colon bacilli being placed under new conditions, undergo modifications as a result of which the so-called typhoid bacillus is evolved, and the serum of the blood becomes capable of causing the agglutination of this particular variety of the colon bacillus. While the above is merely a suggestion as to the relationship of this germ to typhoid fever, and may prove to be entirely incorrect, he has no hesitation in declaring that we do not as yet know positively that Eberth's bacillus produces typhoid fever, and he considers it erroneous and unscientific to assume such a relationship until it is distinctly proven. If this can only be done no one will rejoice more than he will, or be quicker to accept it.

Dr. Victor C. Vaughan, Ann Arbor, says that he accepts unqualifiedly the etiological relationship of the Eberth bacillus to typhoid fever. He believes, however, that there are varieties of this bacillus, and that its cultural manifestations are subject to considerable variations. He believes that the agglutination and reaction show the specific nature of the Eberth bacillus. He does not believe that typhoid fever can be aborted by any known method of treatment after it develops clinically. In the treatment of typhoid fever, he believes in keeping the intestines clean and attending to elimination, but he does not indorse the so-called antiseptic treatment of the disease. He does not believe there is any way of disinfecting the alimentary canal, and even if this could be done, in a case of typhoid fever it probably would not affect the course of the disease, except in so far as the intestinal lesions are concerned. He thinks the experience of intelligent physicians in every part of the world has shown the superiority of the Brand system of treatment in this disease.

Dr. J. S. Cain, Nashville, says typhoid fever is produced by a micro-organism which finds a lodgment on the mucous membrane of the small intestine, soon penetrating into the sub-mucous lymphoid structures and chiefly found at the lower part of ileum. Here the bacilli multiply and manufacture the ptomaine of typhoid. The patient feels the effect of the slowly accumulating toxine and suffers from the prodrome of the attack. The germs themselves cease to be potent factors from the time their poisonous products enter the circulation in sufficient quantity to impress the nerve centers and to cause the obvious phenomena of typhoid fever—that is, after the advent of pyrexia, though they continue to live in various phases, perhaps, for months and even for years. The causative toxine is ultimately eliminated by the emunctories, or, more likely, antidoted by an antitoxine manufactured within the economy. Secondary septic conditions frequently ensue. Hence the disease to be treated is the result of a prostrating poison. If complications arise they must be met. The minute amount of mercury contained in the Woodbridge compound is the only agent therein which could reasonably be accused of having any specific effect. The other ingredients are harmless and worthless. The question of diagnosis is important. Statistic-makers, with a fresh, new hobby, are unreliable, not always from design, but from enthusiasm and preconceived notions. He has but little patience with diagnoses made in some dark laboratory by a person who never saw the given case or, perhaps, any other case. He thinks a clinical diagnosis—which may generally be made after the first week, but not earlier—should be relied upon above all other evidence.

He asserts that much of the testimony published in favor of these commercial schemes and nostrum methods, though made up in the main by good men, are fallacious. The claims put forward in regard to the influence of certain drugs in the alimentary tract are ridiculous. The old remedy, oil of turpentine, he regards as useful—not as an anti-ferment or anti-anything-else, but as a first-class stimulant to the tissues. After giving an initial dose of calomel, he relies upon hygienic measures, proper diet, perhaps diaphoretics and diuretics, and keeping the temperature below the spoilative point by sponging and, when necessary, by cold tub baths. His object is to tide the patient quietly over the period of danger, holding in reserve proper remedies to meet exaggerated symptoms or complications should they occur.

Dr. R. H. Fitz, Boston, accepts, in the light of our present knowledge, the etiological relationship of Eberth's bacillus to typhoid fever. He does not believe that typhoid fever can be aborted by any known method of treatment, and he does not indorse the so-called antiseptic and eliminative plan of treating this disease. He is accustomed to consider as essential in the treatment of typhoid fever the use of cooling baths, a bland and nutritious diet, the maintenance of cleanliness and such medication as is indicated for any complications which may arise.

Dr. Frederick C. Shattuck, Boston, regards the Woodbridge or eliminative treatment of typhoid fever faulty in theory and pernicious in practice. It is based on the assumption that typhoid bacilli are practically confined to the intestinal tract, while it is known that they penetrate the intestine and mesenteric glands, the spleen, the rose spots and the gall-bladder. Sweeping the surface of the intestines cannot influence deeper structures. If diarrhea is desirable in typhoid fever those cases in which diarrhea is a feature should do better than those with constipation. Such, however, is not the experience of the Massachusetts General Hospital, or, so far as he knows, of any other hospital. He believes any doctrine is dangerous which proclaims that typhoid fever may be treated without confining the patient to bed. When typhoid fever kills, it does so either by perforation or by exhaustion, the proportion of the former being estimated at 5 to 10 per cent. Most of us are agreed that we are not as yet acquainted with any therapeutic measures which will abort or very materially shorten the course of the disease. We are, he thinks, unanimous in believing that husbanding the strength from the start, skillful nursing, the judicious use of water—internally and externally— and a wise supervision, meeting indications as they arise, materially modifies the course of the disease and lessens its mortality. He advocates a more liberal diet than is usually allowed, treating the patient rather than the disease, and feeding him according to his digestive power. In addition to the usual liquid foods, he advises ice cream, soft-boiled or raw eggs, egg-nog, minced or scraped beef, the soft part of raw oysters, soft crackers, soft toast, pudding, blanc mange, wine jelly, apple sauce and macaroni.

Dr. A. Jacobi, New York, says, in regard to the most approved treatment of typhoid fever, the food should be liquid, and continued unchanged in character for at least ten days after apyrexia is established. Water should be freely taken. A purgative dose of calomel in the very beginning will act beneficially, but not so after the first week, when diarrhea and hemorrhages may be provoked by it. Constipation requires warm water enemata daily. Cold water and warm water are the best and safest antipyretics. Stress is laid on the value of the latter. Cold bathing is frequently contraindicated. All patients do not bear cold water well and certain complications would preclude its use. Statistics do not speak well for the indiscriminate use of cold water in hospital practice. Warm water bathing at a temperature of 90° to 95° F. is not obnoxious to the same objections, and it will reduce high temperatures. This is simply a fact. Warm bathing should be the principal treatment in typhoid fever, but not to the exclusion of occasional medication. This treatment will always remain appropriate even though our resources should be increased, as we expect, by sero-therapy,—what is to be treated is not the bacillus, but the organism invaded by the bacillus. The clinician should know that bacteriology is an indispensable aid to clinical medicine, but that it is not clinical medicine itself.

Untoward symptoms are to be met by appropriate treatment. Diarrhea, a feeble heart, collapse, insomania and other symptoms may call for special medication. Cardiac stimulants, skillfully applied, are mighty weapons in the hands of the intelligent medical adviser, who need not necessarily limit himself to a few.

Thus, after all, when we shall have found an antitoxin for the typhoid bacillus, we shall still require adjuvant treatment for the typhoid man, woman and child.

Dr. W. P. Northrup, New York, says that his views as to the proper treatment of typhoid fever are absolute quiet in bed, good ventilation, and tubbing when the temperature runs above 103° F. Milk and broth diet, with a return to solid food only after the temperature has been normal for ten days. He has had no experience with the Woodbridge treatment, but it has been tried by others to his satisfaction and found to be of no value whatsoever, and, theoretically, it is utterly unworthy of confidence.

Dr. J. C. Wilson, Philadelphia, writes that the Woodbridge treatment for typhoid fever is a so-called eliminative treatment that not only does not, but cannot eliminate. The statistics used to support the allegations of its promoter were crude and unreliable, and the opportunities afforded him to test his treatment in military service resulted in the final demonstration of the uselessness of his plan.

His own experience leads him to believe that a large proportion of the cases of enteric fever seen in Philadelphia—one of its great strongholds—would terminate in recovery under a pure expectancy, and that a small percentage would terminate in death under any treatment yet devised; there is a third group of cases in which the result is largely influenced by treatment. Up to the present time he knows of no method of dealing with the disease that minimizes the death-rate in this group to the same extent as systematic cold-bathing according to the method of Brand. It is an interesting fact that while the mortality ranges, under other methods of treatment, somewhere between 12 and 18 per cent., the large statistics of this plan by observers in different parts of the world show, with great uniformity, a total average mortality of between seven and eight per cent.

Dr. James Tyson, Philadelphia, has no belief in the so-called eliminative or Woodbridge treatment of typhoid fever, nor in any amendments, modifications or variations of it which have come to his knowledge. Moreover, so far as he has been able to examine into its results, as obtained in the army and navy hospitals during the Cuban war, these results have been in no way better than those obtained by other methods of treatment.

Dr. William Osler, Baltimore, writes as the proper principles of treatment:

First. A careful and thorough system of nursing, to which, as much as to any other single feature, he attributes the comparatively low rate of mortality for a general hospital.

Second—Diet. Milk diluted with lime water and egg albumen form the standard diet of the febrile stage. Artificial foods are rarely ordered.

Third—Hydrotherapy. Either the full tub-bath at 70° F.; or, if occasion requires, ice-cold sponges.

Fourth—Drugs. As a rule, no medicines are used. If the pulse becomes rapid and feeble, alcohol, in the form of good whisky, and strychnine, if necessary, in full doses, are given. No antipyretics and no intestinal disinfectant are employed. Special complications, of course, require and should receive appropriate treatment.

Dr. Frank Billings, Chicago, says the old so-called specific forms of treatment in the shape of bowel antiseptics, like iodine, carbolic acid, calomel, etc., are no longer considered specific by any one but the enthusiastic few, who still treat typhoid fever and not the typhoid patient. No known drug will shorten the duration of the disease. It will run its course in spite of any known treatment. Drug antipyretics, cathartics, especially calomel in large or in small doses, and so-called intestinal antiseptics, have run a more or less unhappy course and are now practised by but few thinking men.

In the early stage a cathartic is useful; prefers calomel or some other mercurial. Hydrotherapy, preferably the Brand bath, or a modified application of the bath, affords better results than any other form of symptomatic treatment, but it should be employed with nice discrimination. The diet should be liquid or semi-solid.

The main principle is to select simple, easily-digested food which will leave but little irritating residue in the bowel. Milk alone is not approved, for it does not agree with all. The drug treatment should consist in the use of remedies indicated in emergencies as they arise. Strychnine and digitalis may be required on account of a feeble heart. Constipation, when it exists, may be overcome by simple laxatives, though enemas daily, or every second day, usually suffice. A plentiful amount of water, as a diluent drink, should be used. He has no confidence whatever in the Woodbridge treatment, neither has he confidence in nor does he use the so-called antiseptic forms of treatment.

Dr. N. S. Davis, Jr., Chicago, relies in the management of typhoid fever upon a modified Brand treatment, careful regulation of the diet, good nursing, thorough ventilation, and the maintenance of good action of the kidneys.

Dr. Robert H. Babcock, Chicago, is not a believer in the Woodbridge method of treating typhoid fever, and he has had no experience with any other antiseptic plan of treatment. He believes that the best results are furnished by keeping the intestinal tract as clean as possible through the early administration of calomel and the subsequent daily use of colonic flushing, by reduction of the temperature by means of cold—preferably the Brand method—by a dietary consisting of perfectly bland foods but not exclusively of milk, and by the treatment of complications as they arise. It is essential to supply the patient with a liberal quantity of fluid for the purpose of flushing the kidneys, and colonic lavage is largely a benefit in this way.

Dr. Henry B. Favill, Chicago, writes that the Woodbridge treatment has always seemed to him a misconception, and hence he has not given it that careful observation and trial that he would give something that appealed to him.

Dr. G. Baumgarten, St. Louis, says he has never become familiar with the "Woodbridge treatment," and has never experienced the want of it. His treatment of typhoid fever is almost exclusively by nursing and bathing, with very little medicine.

Dr. John G. Cecil, Louisville, states briefly, from what he understands and believes to be the true etiology and pathology of typhoid fever, he has not thought it possible, by any plan of treatment, to abort or very much modify its course, as the typhoid germ has pervaded the system before a definite diagnosis can be made. He prescribes an initial mercurial cathartic and, later, salol or similar drugs which he thinks improves the condition of the alimentary canal.

Dr. H. H. Levy, Richmond, says the patient should be kept in bed from the beginning and for ten days after the cessation of fever. The diet should be liquid and easily digestible. Allows an abundance of pure water, a full dose of calomel at first, and guaiacol carbonate to prevent putrefaction in the bowels. Strychnine and other heart tonics and stimulants are required. Cold water, applied preferably with some friction, for hyperpyrexia, and an ice bag to the head for headache or delirium in private practice, but when practicable, tub-bathing is the ideal method. Enemas to overcome constipation once daily. Insures activity of kidneys.

Dr. Joseph W. Irwin, Louisville, says: The prime indication in the treatment of disease is to remove the cause, but in typhoid fever no medicine will do this, so that our chief reliance must be on diet and nursing. He does not believe that the course of the disease can be shortened by drugs. Discards, as of no consequence, the Woodbridge claims. Advises a cathartic dose of calomel at the outset, and in uncomplicated cases, a mild laxative—preferably castor oil—may be indicated at intervals during the attack. Remedies should be addressed to symptoms as they appear, which are usually severe in proportion to the gravity of the changes occurring in the tissues. Insomnia may be met by opiates. Tympanitis, by castor oil and turpentine, sometimes by opium. Alcohol should be commenced before cardiac weakness is prominent and administered according to the effect on the circulation. Hyperpyrexia requires sponging with water and alcohol, or, under certain conditions, the Brand system of bathing. Besides stimulants, cardiac tonics are generally indicated. Absolute rest is essential, a digestible, liquid diet, regulated in amount by the digesitive power of the individual. The abundant use of water by the mouth for its flushing effect upon the kidneys, good ventilation and disinfection of the dejections constitute the essentials of judicious treatment.

For hemorrhage and perforation, advises opium with ice to abdomen, and laparotomy, respectively.

Dr. Derring J. Roberts, Nashville, states that the so-called eliminative treatment of enteric or typhoid fever is not, to his mind, at all satisfactory. The germs of the disease and their toxins are beyond the reach of either antitoxins or eliminatives long before a correct diagnosis can be made. His reliance is upon the free use of water, internally and externally—the Brand method or its most readily obtainable analogue—careful attention to diet, nursing, cleanliness of person and surroundings, with supportive measures and stimulants when indicated. Do not exhaust the patient but sustain him and let the disease exhaust itself.

Dr. J. S. Todd, Atlanta, believes that typhoid fever is an acute, infectious, self-limited disease, and that any medication designed to abort its course is not only useless, but, if pushed, becomes positively harmful. The recently exploited views concerning elimination are but a revival of long discarded theories which held full sway when the lancet, purgatives, emetics and diaphoretics were vigorously resorted to. Remembering the length and the construction of the intestine, who can seriously believe that it is possible to disinfect it with a few grains or a few drops of any antiseptic? and how shall the germs in other organs be reached?

When the temperature exceeds 102° F., he uses the tub bath, with water to suit the feelings of the patient. When this is impracticable, he substitutes sponging with cold or warm water, employing friction before, during and after the bath. Ice to the head, nucha and right iliac fossa, for pain, restlessness or hemorrhage, and large enemas of ice water to limit bleeding and reduce heat. In exceptional instances, gives drug antipyretics, always guarded by strychnine, camphor, caffeine or alcohol. The bowels should be kept open for the first few days by cathartic doses of calomel. Oil of turpentine is serviceable for its antiseptic, diuretic, carminative and heart-tonic effects and, in the language of the great John Hunter, it is "the best, if not the only true, internal hemostatic."

Liquid diet is imperative during the attack and for ten days after the fever has ceased. Alcohol is required in many, but not in all cases.

Dr. Charles G. Giddings, Atlanta, believes the "Woodbridge treatment" in typhoid fever is not only irrational in theory, but is absolutely dangerous in practice. He classes it with the numerous fads that seem to belong to this age of medicine. His treatment is a calomel purge at the outset, to be followed during the course of the disease by enemata when necessary. Liquid diet, preferably milk. Stimulants, usually after the first week. Has never been satisfied that intestinal antiseptics play an important part. On account of the damage to the blood and nervous system by continued high temperature, endeavors to hold it in check, preferably with phenacetine or baths, or both. He has never seen an attack of typhoid fever aborted, and knows of no specific treatment for the disease.

Dr. C. D. Hurt, Atlanta, says, as initial treatment in typhoid fever, he gives a mercurial cathartic, either the proto-iodide or the mild chlorid, and quinine for the first three or four days to differentiate if malaria is suspected. He seeks to control high temperature with guaiacol carbonate, salol and general baths. Regulates functions of the digestive organs with antiseptics, mercury in small doses, salol, naphthol and oil of turpentine. Relieves tympanites and secures fecal evacuations with enemas of turpentine and warm water. Nourishes with milk and meat extracts, avoiding solid food. Stimulates with strychnine and alcohol, when needed, to sustain the vital forces. Regards the Woodbridge treatment as complicated and impractical. Elimination by purging is disappointing, is too irritating and exhausting.

Dr. McHatton, Macon, is of the opinion that we have no line of treatment of the so-called eliminative or antiseptic kind which has any influence on the duration or the severity of the attack. Every case of typhoid fever should be treated on its merits. He combats unfavorable symptoms as they arise. Gives close attention to hygiene and diet. Insists on good nursing. Occasionally, the coal tar antipyretics, very moderately and cautiously, are serviceable, but treats simple, uncomplicated cases often without a single dose of medicine.

Dr. St. John B. Graham, Savannah, advises a liquid diet. There is no specific remedy for typhoid fever, and the expectant treatment yields the best results.

My own experience, gentlemen, sustains the views, as to the general principles of treatment, unanimously expressed in the foregoing paragraphs, and my deliberate judgment indorses these principles, with the light now before us, as essentially sound, as eminently safe and as entirely trustworthy.

